# Validation of CRISPR activation system in *Aedes* cells using multicistronic plasmid vectors

**DOI:** 10.3389/fbioe.2023.1142415

**Published:** 2023-04-19

**Authors:** Vijeta Jaiswal, Sara Ashok Varghese, Sanjay Ghosh

**Affiliations:** Institute of Bioinformatics and Applied Biotechnology, Bengaluru, Karnataka, India

**Keywords:** dCas9-VPR, CRISPR activation, *Aedes* mosquito, multicistronic vectors, C6/36 cells, transcription activation

## Abstract

*Aedes* mosquitoes transmit several pathogens including flaviviruses to humans which result in high morbidity and mortality. Owing to adaptability and climate change, these mosquito vectors are predicted to establish in new geographical areas thus exposing larger populations to the risk of infection. Therefore, control of *Aedes* vector is necessary to prevent disease transmission. Recently, genetic approaches to vector control have shown promise; however, the tools and methods for manipulating the mosquito genome are rather limited. While CRISPR-Cas9 system has been adapted for gene editing purposes in *Aedes* mosquito, the dCas9-based transcription control of genes remain unexplored. In this study we report implementation of the CRISPR activation system in *Aedes* cells. For this we designed, constructed and tested a bi-partite plasmid-based strategy that allows expression of the dCas9-VPR and targeting guide RNA together with a reporter cassette. Quantitative analysis of the fluorescent reporter gene levels showed a robust over-expression validating CRISPR activation in *Aedes* cells. This strategy and the biological parts will be useful resource for synthetic transcription factor-based robust upregulation of *Aedes* genes for application of synthetic biology approaches for vector control.

## Introduction

*Aedes* mosquitoes (*Aedes aegypti* and *Aedes albopictus*) are principal vectors for transmitting several arthropod borne viral diseases to humans which includes dengue, zika, yellow fever and chikungunya. These diseases disproportionately affect the poorest populations causing substantial morbidity and mortality. Although currently distributed throughout the tropical and subtropical regions of the world (Kraemer et al., 2015), *Aedes* mosquitoes are predicted to invade new geographical areas owing to higher adaptability and climate change (Iwamura et al., 2020). Spread of mosquito vectors poses threat of spreading of mosquito borne diseases beyond the tropical regions of the world. Over the past decade, the re-emergence of these viral diseases and epidemics has been a cause of global concern. In absence of effective vaccines or therapy, control of the mosquito vector is vital to reduce the disease burden and protect people from infection. While traditional interventions to control mosquito population have limited success, genetic approaches have shown promising results (Wang et al., 2021). With increasing availability of the “omics” data, it is now feasible to gain mechanistic insights into the role of host factors in virus life cycle and develop rational genetic strategies for vector control and disease management. However, the genetic tools and technologies to manipulate the mosquito genome efficiently are lacking.

The CRISPR-Cas9 system, repurposed from the bacterial adaptive immune system, is a two component RNA-directed DNA cleavage system that consists of a Cas9 endonuclease and a non-coding single guide RNA (sgRNA) containing a 20 nt DNA targeting sequence (Jinek et al., 2012). The Cas9-sgRNA complex binds to the complimentary strand of DNA adjacent to a conserved three nucleotide protospacer adjacent motif (PAM) and creates a blunt-ended double-stranded break that results in indel (insertion-deletion) mutations due to imperfect repair by the non-homologous end joining (NHEJ) DNA repair pathway. In addition to DNA editing, nuclease deficient “dead” Cas9 (dCas9) has been fused with effectors and used as synthetic transcription factors (La Russa and Qi, 2015). For example, fusing dCas9 protein with either transcription activators (VP16, p65, rta, VPR, *etc.*) or repressor (KRAB, MeCP2, Mxi, *etc.*) domains led to ectopic activation (CRISPR activation system; CRISPRa) or repression (CRISPR interference system; CRISPRi) of the target genes, respectively (Larson et al., 2013; Gilbert et al., 2014; Ghosh et al., 2016; Liao et al., 2017). In addition, diverse CRISPR systems have been adapted successfully for targeting DNA and RNA for various applications (Pickar-Oliver and Gersbach, 2019). While CRISPR-based strategies have been used to modify the *Aedes* genome (Kistler et al., 2015; Li et al., 2017; Liu et al., 2019; Rozen-Gagnon et al., 2021), its use in modulating gene expression using dCas9-based systems has not been explored in *Aedes* cells.

Here, we report construction of a versatile plasmid-based system that allows rapid testing of transcription upregulation of genes in *Aedes* cells. Using a fluorescent reporter based assay we demonstrate a robust gene over expression, thus validating the CRISPRa system in the mosquitoes.

## Methods

### Plasmid construction

The pJVG1 plasmid, constitutively expressing dCas9-VPR in *A. albopictus* C6/36 cell line was generated using Gibson assembly method. Briefly, the dCas9-VPR encoding sequence was amplified from the Addgene plasmid #63801 (a gift from GM Church; Chavez et al., 2015) whereas the rest of the plasmid backbone was amplified from PUb-HeFSpCas9 plasmid (unpublished) using primers SB1_F/R and SB2_F/R. To generate the fluorescent reporter vector pJVG2, the RNA polymerase III promoter 7SK (AALF029648) and sgRNA scaffold along with poly-T terminator sequences (Anderson et al., 2020) were synthesized as gene fragments from Twist Biosciences. The 20 nt tetracycline operator (tetO) targeting sequence was designed using CHOPCHOP tool (Labun et al., 2019). The 7X-tetO repeat cassette was amplified from the Addgene plasmid #92099 (a gift from Y Doyon; Dalvai et al., 2015) and the UbL40 promoter was amplified from *Aedes* genomic DNA. The mCherry-T2A-pac-SV40polyA cassette along with the entire vector backbone was amplified from AaU6-sgGFP-PUB-mCherry-T2A-pac plasmid (unpublished) using primers SB5_F/R and SB6_F/R.

To construct pJVG-T2A and pJVG-dT2A, the PUb promoter was amplified from *A. aegypti* genomic DNA using primer pairs SB7_F and SB7_R while the mCherry and eGFP genes were amplified from Addgene plasmid #79124 (a gift from WA Lim; Morsut et al., 2016) and #64709 (a gift from K Musunuru, unpublished), respectively, using primers SB9_F/R for mCherry and SB8_F/R for eGFP. The T2A and dT2A sequences were derived from Addgene plasmid #84743 (a gift from F Zhang; Zetsche et al., 2017) and #32426 (a gift from JD Sutherland; González et al., 2011), respectively. The vector backbone containing SV40 polyA, ampR gene and ori sequences was amplified from Addgene plasmid #78899 (a gift from G Church; Chavez et al., 2016) using primers SB6_F/R.

To generate plasmids PUb-eGFP and UbL40-eGFP, the promoters were amplified from *Aedes* genomic DNA using primers SB7_F/R and SB10_F/R while the polyhedrin promoter was obtained from pFastBac vector for polyhedrin-eGFP and cloned into pJVG-T2A vector using Infusion HD cloning kit (TakaraBio #102518) replacing the mCherry-T2A cassette.

Polymerase chain reactions (PCRs) were performed using Q5 High-Fidelity polymerase (NEB #M0491) and Phusion High-Fidelity polymerase (NEB #M0530). The PCR amplicons were purified using FavorPrep Gel/PCR purification kit (Favorgen #FAGCK001) following manufacturer’s protocol. The purified fragments were assembled using Gibson assembly cloning kit (NEB #E5510) and In-fusion HD cloning kit (TakaraBio #102518), and the reaction transformed into chemically competent *E. coli* DH5 alpha cells following standard protocols. Recombinant plasmids were isolated using FavorPrep Plasmid DNA Extraction mini kit (Favorgen #FAPDE300), digested with restriction enzyme for confirmation and validated by Sanger sequencing (Eurofins Genomics). All oligonucleotides were procured from Eurofins Genomics and Merck. The list of primers is provided in Supplementary Table S1. Detailed methods are available upon request.

### Cell line transfection and fluorescence microscopy

*Aedes albopictus* C6/36 cell line was purchased from National Center for Cell Sciences (NCCS), Pune, India. Cells were grown in Minimum Essential Medium Eagle media (Merck #M5650) supplemented with 10% fetal bovine serum (MP Biomedicals #29101), 1X Antibiotic-Antimycotic (Gibco #5240062) and 1X Glutamax (Gibco #35050061) at 28°C with 5% CO_2_.

pJVG1 and pJVG2 plasmids were co-transfected in 1:1 ratio (6 µg total) in C6/36 cell line using Xfect reagent (TakaraBio #631317) according to manufacturer’s protocol. Cells were seeded at ∼ 60% confluency in a 6-well cell culture multi dish (Thermo #140675) and complexes formed using 0.3:1 transfection reagent to plasmid ratio. The transfection mix was removed after overnight incubation and replaced with fresh complete media. 48 h post-transfection, cells were imaged using EVOS FL Auto 2 Cell Imaging System (Thermo Scientific). Post-imaging, cells were harvested, washed once with 1X PBS and samples collected for Flow cytometry and RT-qPCR analysis.

### Flow cytometry

Flow Cytometry analysis of the live cells was performed using Beckman Coulter Cytoflex LX flow cytometer equipped with 488 nm and 561 nm laser. Fluorescence was detected in green (510–540 nm) and red (660–690 nm) channels. Cells expressing a single fluorescent reporter (pJVG2) or untransfected cells were kept as control. All samples were analyzed for 10,000 events. Data from two biological replicates was analysed and plotted using the CytExpert software 2.3.

### RT-qPCR

RNA isolation was performed using Nucleospin RNA kit (Macherey-Nagel #740955) as per the manufacturer’s protocol. cDNA synthesis was performed using Primescript IV 1st strand cDNA synthesis mix (TakaraBio #6215A) according to the manufacturer’s protocol. RT-qPCR was carried out using PowerUp SYBR green master mix (Thermo Scientific #A25742) on StepOne Real Time PCR system (Applied Biosystems). The final volume for each reaction was 10 µL with gene specific primers (SB12_F/R for mCherry and SB13_F/R for PGK) and 1 µL of total cDNA (diluted 1/10th). All samples were in triplicates. The thermal cycling was initiated at 50°C for 2 min followed by 95°C for 2 min as initial denaturation. The cycling reaction was kept at 95°C for 3 s followed by 60°C for 30 s for 40 cycles. Dissociation curve analyses were carried out at the end of each run. Data from two biological replicates was analysed using ΔΔCt method and standard error of mean was plotted.

### SDS-PAGE and immunoblotting

C6/36 cells were transfected with pJVG-T2A and pJVG-dT2A plasmids. 48 h post-transfection, cells were pelleted and washed with 1X PBS followed by lysis in SDS sample loading buffer and heating at 95°C. The lysates were clarified by centrifugation at 12,000 x*g* for 10 min and supernatant collected in a separate tube. Equal volumes of the samples were loaded in duplicate along with Puregene protein dual color standards (Genetix # PG-PMT2922) on a 12% SDS-PAGE gel and electrophoresed in 1X SDS Running buffer in TetraCell apparatus (BioRad) according to manufacturer’s protocol. Following electrophoresis, the protein samples were transferred onto a PVDF membrane (BioRad #162–0177) following the manufacturer’s protocol. Post-transfer, the blot was cut into two and blocked using 5% BSA-TBST solution for 30 min at room temperature followed by incubation with rabbit anti-GFP antibody (1:1000, CST #2956S) and rabbit anti-mCherry antibody (1:1000, Millipore #AB356482) separately. The blots were washed thrice for 5 min each with 1X TBST buffer and incubated with HRP-conjugated anti-rabbit IgG secondary antibody (1:2000, CST #7074S). The blots were then washed thrice for 5 min each with 1X TBST buffer and developed with Clarity Western ECL substrate (Bio-Rad #1705061). Imaging was performed using ImageQuant LAS 500 (GE Healthcare Bio-Sciences AB). After imaging, the blots were incubated with Western Blot Stripping Buffer (Takara Bio #T7135) for 5 min and washed extensively with 1X TBST. Following this, the blot was blocked with BSA-TBST solution as above and incubated with mouse anti-actin antibody (1:1000, Invitrogen #MA1-744), washed with 1X TBST and developed using HRP-conjugated anti-mouse IgG secondary antibody (1:2000, CST #7076) as described above.

## Results and discussion

**FIGURE 1:**
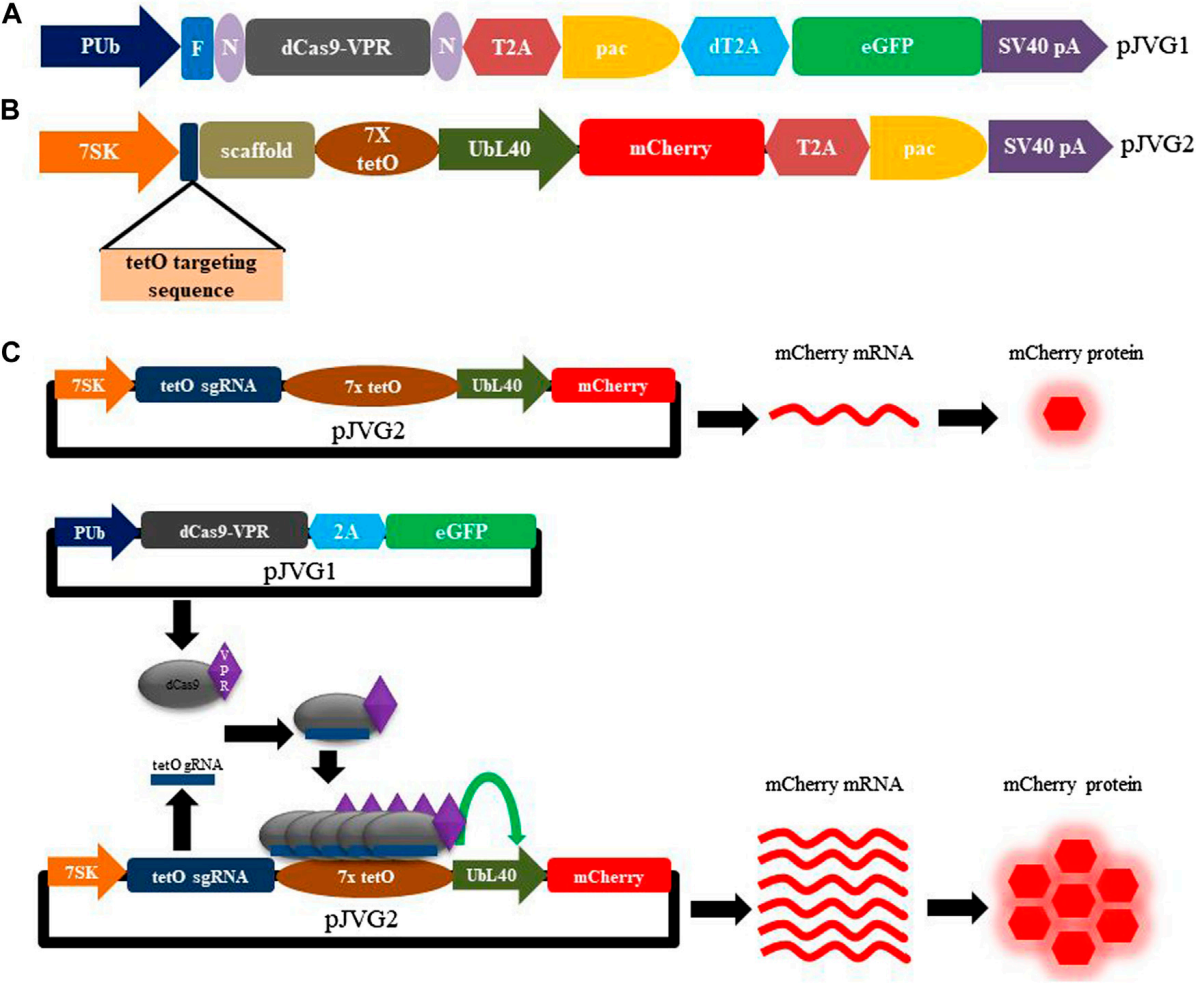
Design of the dCas9-VPR assay plasmids and functional validation of CRISPRa activity in *Aedes* cells. Schematic diagram showing the design of pJVG1 **(A)** and pJVG2 **(B)** plasmids. In pJVG1, the RNA polymerase II promoter PUb regulates the expression of dCas9-VPR, puromycin acetyltransferase (pac) and eGFP which are separated by 2A peptides (T2A and dT2A). The pJVG2 plasmid contains dual expression cassettes wherein the tetO targeting guide RNA and scaffold sequences are transcribed by the RNA polymerase III promoter 7SK while the mCherry and puromycin acetyltransferase (pac) genes, separated by the T2A peptide encoding sequence, are under the control of RNA polymerase II promoter UbL40. Seven copies of the tetracycline operator (tetO) sequence is appended at the 5′end of the UbL40 promoter to facilitate dCas9-VPR recruitment. **(C)** Overview of the CRISPR activation assay using dCas9-VPR in *Aedes* cells. The assay consists of transfecting either pJVG2 plasmid alone or together with pJVG1 plasmid. In absence of plasmid pJVG1, the mCherry mRNA is produced from plasmid pJVG2 at the basal level by the UbL40 promoter which is translated to the corresponding red fluorescent protein. However, co-transfection of pJVG1 and pJVG2 plasmids leads to formation of functional dCas9-VPR-tetO guide RNA complexes which are recruited to the tetO sequences located upstream of the UbL40 promoter resulting in an increased transcriptional activity of the promoter. This results in an increased production of mCherry mRNA and protein which is quantified by RT-qPCR and flow cytometry analysis, respectively. N, NLS sequence, F, FLAG epitope tag, pA, polyadenylation sequence.

We set out to establish the CRISPR activation (CRISPRa) system to over-express genes in the *Aedes* mosquito cells. To implement CRISPRa, we chose dCas9 fused with VPR (VPR is a tripartite activator composed of the VP64, p65, and Rta domains) as transcriptional activator (Chavez et al., 2015) and designed a bi-partite plasmid system for functional evaluation of dCas9-VPR activity using fluorescent reporter assay in *A. albopictus* C6/36 cells (Figures 1A, B). In this scheme, one of the plasmid (pJVG1) encodes the effector protein dCas9-VPR while the other plasmid (pJVG2) transcribes the 20 nt non-coding sgRNA targeting a heterologous sequence (tertracycline operator sequence from bacteria; tetO). Upon co-transfection, the dCas9-VPR-sgRNA complex is formed in the cells and binds the tetO sequence positioned upstream of a RNA polymerase II promoter on pJVG2 plasmid resulting in transcription upregulation of the fluorescent protein mCherry whose levels can be determined quantitatively (Figure 1C).

The dCas9-VPR encoding plasmid pJVG1 is designed to consist of a multicistronic expression cassette with dual selection markers. As shown in Figure 1A, the dCas9-VPR gene, flanked with NLS sequences and tagged with FLAG epitope at the N-terminal end, is followed by the puromycin acetyltransferase (*pac*) and eGFP encoding gene. The expression of this cassette is under the control of the strong constitutively active *Aedes* PUb promoter and SV40 polyadenylation sequence. This architecture allows antibiotic as well as fluorescence-based selection of the transfected cells for transient cell-based assays. In addition, to generate stable cell lines rapidly and efficiently, the transfected cells can be sorted by FACS analysis followed by incubation with puromycin. Finally, the eGFP fluorescence in the transfected cells is indicative of dCas9-VPR expression.

**FIGURE 2:**
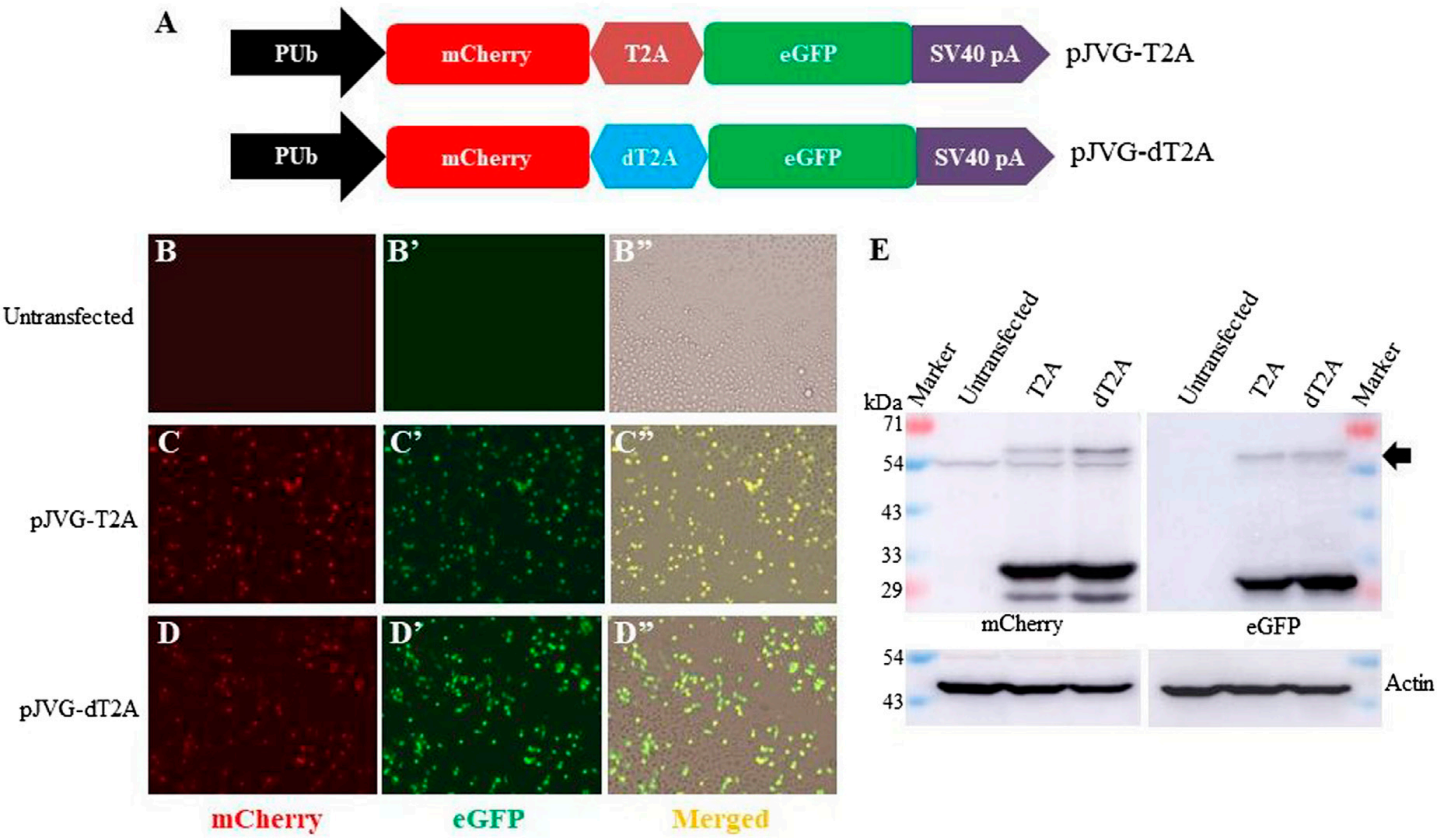
Efficiency of T2A peptides in *Aedes* cells. **(A)** Schematic diagram showing the design of dual fluorescent reporter cassettes to evaluate T2A (plasmid pJVG-T2A) and dT2A (plasmid pJVG-dT2A) activity. **(B-D)** Fluorescence micrograph of the cells transfected with plasmids as shown on the left of the panels. The untransfected cells **(B-B”)** were used to determine the baseline for imaging mCherry and eGFP channels, respectively. **(E)** Western blotting analysis of the extracts prepared from the transfected cells showing presence of processed mCherry and eGFP proteins. The blot was probed with anti-mCherry antibody (left panel) and anti-GFP antibody (right panel). Both blots were subsequently developed using anti-actin antibody. The arrow marks the position of the unprocessed mCherry-eGFP fusion protein.

Our vector design allows transcription of a single mRNA encoding simultaneous co-expression of the dCas9-VPR, puromycin acetyltransferase and eGFP proteins. To separate individual gene products, viral 2A peptides were chosen which mediate peptide cleavage due to ribosome skipping between a conserved glycine and proline residues at the C-terminus of the 2A element (Ryan and Drew, 1994). Although 2A peptides have been used to derive multiple proteins from a single transcript (Liu et al., 2017), the efficiency of peptide cleavage varies in different cell types and organisms (González et al., 2011; Daniels et al., 2014; Souza-Moreira et al., 2018). Importantly, the efficiency of 2A peptides in co-translational cleavage of proteins in *Aedes* has not been determined. Since our plasmid design requires 2A peptides (two in pJVG1 and one in pJVG2), we tested the efficacy of T2A and dT2A elements in *Aedes* cells. For this we generated a bi-cistronic reporter construct wherein the fluorescent proteins eGFP and mCherry are divided by either T2A or dT2A sequences (Figure 2A). Fluorescence microscopy analysis of the transfected cells showed green (eGFP) and red (mCherry) signals (Figure 2B–D”) showing production of functional proteins. To ascertain if the cleavage is efficient, Western blotting was performed using the extracts prepared from the transfected cells. As shown in Figure 2E, both proteins are cleaved efficiently in presence of T2A and dT2A peptides, thus validating their function in *Aedes* cells. Both T2A and dT2A sequences were incorporated in the DNA vectors for the dCas9-VPR assay design.

**FIGURE 3:**
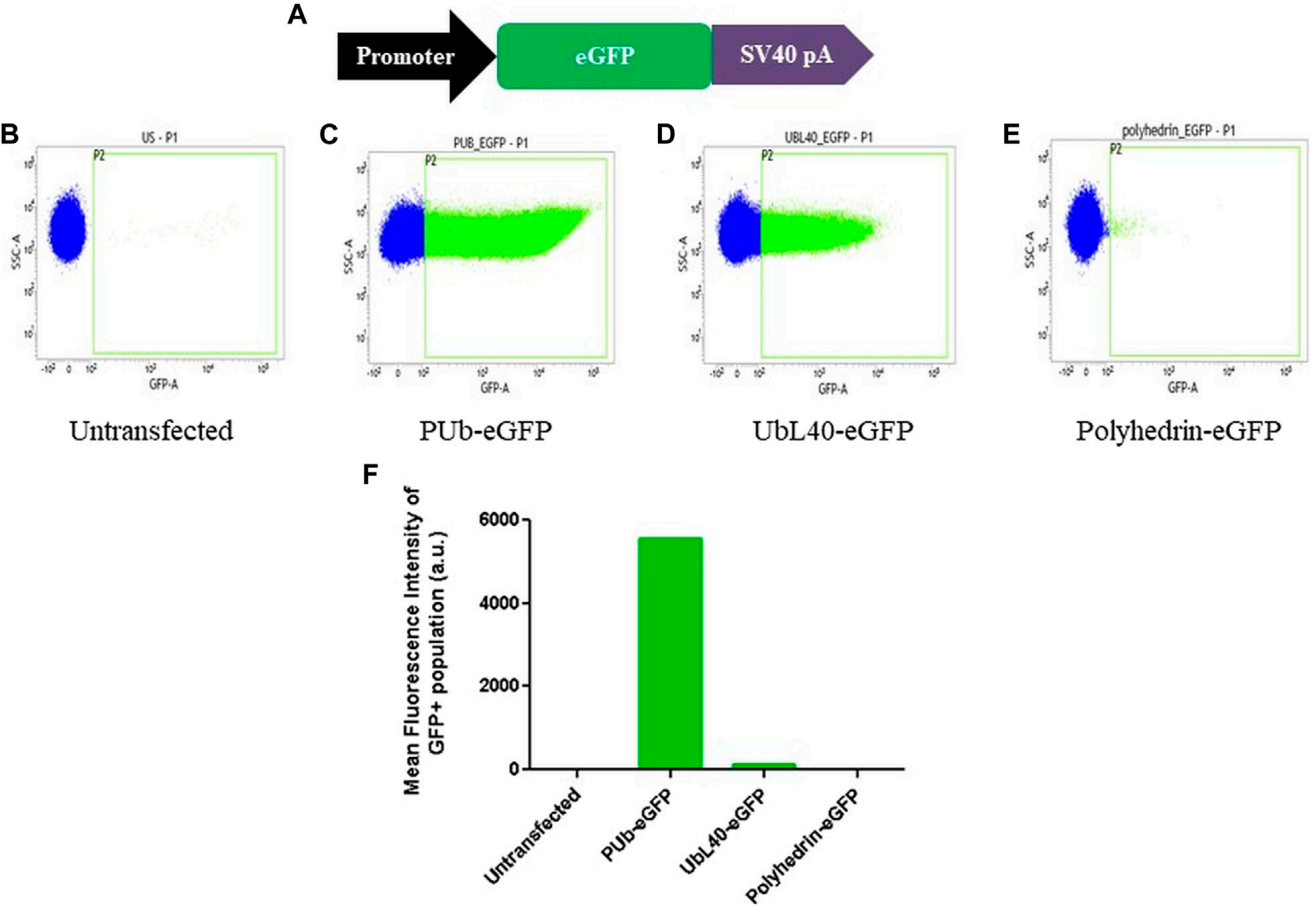
Evaluation of promoter activity in *Aedes* cells. **(A)** Schematic diagram of the design of fluorescent reporter cassettes to evaluate promoter activity. **(B–E)** Flow cytometry analysis of the untransfected **(B)** cells and cells transfected with eGFP gene under the control of PUb **(C)**, UbL40 **(D)** and polyhedrin **(E)** promoters. The histogram plot showing the mean fluorescence intensity of the GFP positive cells is shown in **(F)**.

We next designed the transfection vector pJVG2 to express the guide RNA and the fluorescent reporter cassette (Figure 1B). Recruitment of dCas9-VPR protein upstream of a promoter leads to transcription upregulation of the genes regulated by the promoter (Chavez et al., 2015). To accomplish this, we reasoned that the recruitment of dCas9-VPR fusion protein at the 5’ end of a promoter with weak or low basal activity but high inducibility is desirable. However, a comparative analysis of the strength of promoter activity in *Aedes* cells is not available. Therefore, we performed a fluorescent reporter based promoter activity assay wherein the eGFP coding region was cloned downstream of three constitutive promoters (Figure 3) and the DNA constructs used to transfect *Aedes* C6/36 cell line individually. Analysis of the eGFP signal strength by flow cytometry analysis revealed that, as compared with the highly active PUb (polyubiquitin) promoter, UBL40 (ubiquitin fusion ribosomal protein L40) promoter showed lower activity while the baculovirus polyhedrin promoter was least active in *Aedes* cells. We chose to express the mCherry reporter gene under the control of UbL40 promoter to generate the reporter cassette in pJVG2.

To engage dCas9-VPR protein upstream of the pUBL40, we selected the conserved 19 bp tetracycline operator (tetO) sequence which is derived from the tetracycline operon in bacteria. This sequence is heterologous to *Aedes* cells and likely to have low off-target effects with respect to dCas9-VPR binding. To implement the synergistic effect of dCas9-VPR activity, seven copies of the tetO sequence was cloned at the 5’ end of the UbL40 promoter in pJVG2 (Figure 1B). In addition, the guide RNA expression cassette consisting of the 20 nt tetO targeting sequence along with an optimized guide RNA scaffold sequence was placed under the control of the RNA polymerase III promoter 7SK which showed constitutive and robust expression in *A. albopictus* cells (Anderson et al., 2020).

**FIGURE 4:**
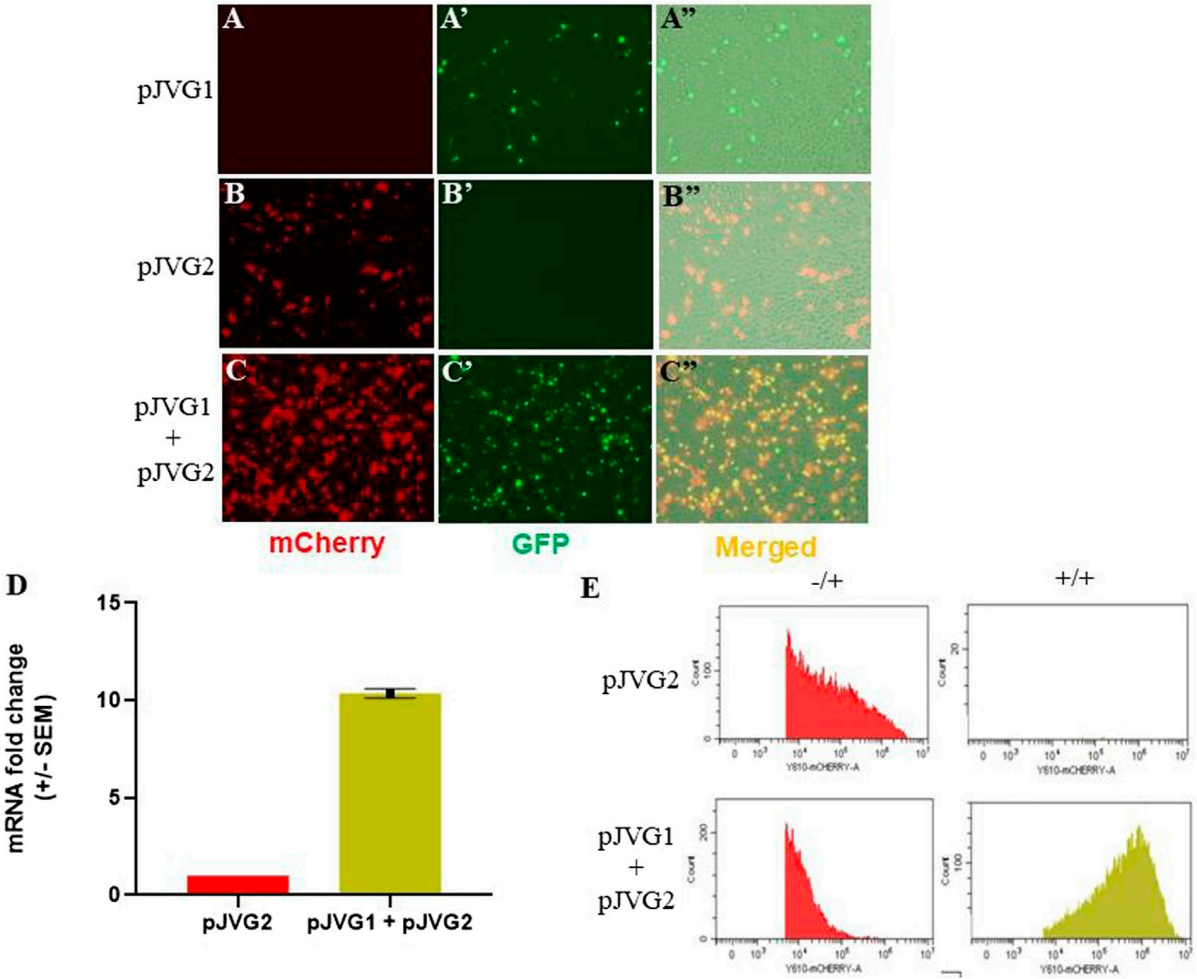
Functional validation of CRISPR activation in *Aedes* cells. C6/36 cells were transfected with either pJVG1 **(A-A”)**, pJVG2 **(B-B”)** or pJVG1 and pJVG2 plasmids simultaneously **(C-C”)** and imaged using fluorescence microscopy 48 h post-transfection. Compared with dCas9-VPR alone, an increased intensity of the mCherry fluorescent signal was observed in cells expressing dCas9-VPR and tetO guide RNA indicating increased mCherry protein levels. **(D)** RT-qPCR analysis showing fold change in expression of mCherry mRNA in cells transfected with pJVG2 alone and co-transfected with pJVG1 and pJVG2. The data is shown for two independent biological samples. **(E)** Histogram plot showing the intensity of mCherry protein in cells transfected with pJVG2 (upper panel) and pJVG1 together with pJVG2 (lower panel). The plots in the first column (−/+) are derived from the quadrant dot plot showing the population of only mCherry positive cells. The plots in the second column (+/+) is derived from the same dot plot showing the population of mCherry and eGFP positive cells.

The plasmids pJVG1 and pJVG2 (Figures 1A, B) were assembled using Infusion DNA assembly method and validated by Sanger sequencing. To evaluate dCas9-VPR function as a transcription activator, *A. albopictus* C6/36 cells were transfected with the dCas9-VPR expressing plasmid pJVG1, the sgRNA-reporter containing plasmid pJVG2 or both pJVG1 and pJVG2 together. Cells were imaged 48 h post-transfection under a fluorescent microscope and samples collected to quantify the levels of mCherry mRNA and protein using RT-qPCR and flow cytometry analysis, respectively. The dCas9-VPR gene was expressed in *Aedes* cells as inferred from the eGFP expression (Figure 4A'). As shown in Figure 4B, we observed substantial mCherry expression in cells transfected with the pJVG2 plasmid. However, the intensity of mCherry appeared to be higher in the cells co-transfected with pJVG1 and pJVG2 suggesting increased protein levels (Figure 4C). Indeed, qPCR analysis revealed a 10-fold increase in the mCherry mRNA levels in the cells co-expressing dCas9-VPR and tetO sgRNA as compared with cells expressing the reporter construct alone (Figure 4D). This data demonstrates transcriptional activation of mCherry gene in a dCas9-VPR dependent manner. Furthermore, flow cytometry analysis shows a similar 10-fold increase in mCherry signal intensity in the co-transfected cells (Figure 4E, Supplementary Figure S1) which co-relates well with the increased mRNA levels. Taken together, this provides the proof of concept of dCas9-VPR based gene over-expression in the *Aedes* cells.

In this study we describe the generation of plasmids designed for evaluation of CRISPR activation system in *Aedes* cells. The architecture of both plasmids allows dual selection of the co-transfected cells using fluorescence-based assays and antibiotics-based selection. These features permit rapid generation of stable cells lines. In addition, we show the efficacy of viral 2A peptide sequences in *Aedes* cells for the first time which should facilitate co-expression and cleavage of multiple gene products from one construct. Finally, our results demonstrate functional validation of dCas9-VPR activity in transcriptional upregulation of genes in *Aedes* cells. This proof of concept study paves way for gain-of-function CRISPRa genetic screens aimed to identify mosquito host factors and gain mechanistic insights into vector competence. Moreover, the DNA vectors generated in this study are an important resource for up regulating anti-viral host factors in *Aedes* mosquitoes for vector control and disease management. In the future, CRISPR activation can be used to engineer mosquitoes with desirable traits aimed to minimise the impact of vector-borne diseases in humans (Buchman et al., 2020). However, the implementation of this technology must comply with the appropriate regulatory framework and involve public engagement (Scudellari, 2019; World Health Organization, 2021; Meghani, 2022).

## Data Availability

The original contributions presented in the study are included in the article/Supplementary Material, further inquiries can be directed to the corresponding author.
