# Supplementary material for: Validation of CRISPR activation system in Aedes cells using multicistronic plasmid vectors
Source: Front Bioeng Biotechnol. 2023 Apr 19;11:1142415. doi: 10.3389/fbioe.2023.1142415 (PMC10155059; doi:10.3389/fbioe.2023.1142415)
Supplement: Supplementary file 1 [file DataSheet1.pdf]

Supplementary Material

Validation of CRISPR activation system in *Aedes* cells using multicistronic plasmid vectors

Vijeta Jaiswal[1], Sara Ashok Varghese[1,2], Sanjay Ghosh[1,*]

[1] Institute of Bioinformatics and Applied Biotechnology, Biotech Park, Electronic City Phase I, Bengaluru, Karnataka, 560100, India

[2] Current Address: Syngene International Limited, Biocon Park , SEZ, Bommasandra Jigani Link Road, Phase-IV, Bommasandra Industrial Area, Bengaluru, Karnataka, 560099, India

[*] Correspondence:

Sanjay Ghosh
ghosh_s@ibab.ac.in

Supplementary Table S1:

Primer	Seq (5' to 3')
SB1_F	AAGGTCGGTATCCACGGAGTCCCAGCAGCCATGGACAAGAAGTACTCCATTGG
SB1_R	GCCGGCCTTTTTCGTGGCCGCCGGCCTTTTGGATCCAAACAGAGATGTGTC
SB2_F	AAAAGGCCGGCGGCCACG
SB2_R	GGCTGCTGGGACTCCGTGGATACC
SB3_F	ACCGTACACGCCTAAAGC
SB3_R	ACTCCTTGATGATGGCCATGGTGGCGGTGAATTCTCCAGGCG
SB4_F	ATAGGGAGTAAACTCGAGTATGTCGAACCTTGCATGCGTGCAC
SB4_R	ACTCCTTGATGATGGCCATGGTGGCCTTGGATCAGTCTGTGGAAAAGTC
SB5_F	TTTTCTTCCATTTCAGGTGTCGTGAGCCACCATGGCCATCATCAAG
SB5_R	GCCCTCTTAATTAAGTCGGTCAAGCCGCTCGGTCGTTCGGCTG
SB6_F	TGTCCAAACTCATCAATGTATCTTACCGTTTAAACTACGCGTAATTCAA
SB6_R	TCTTCCGCTTCCTCGCTC
SB7_F	TATCTTTACATGTAGCTTGTGCAT
SB7_R	ATTCGTTGAAATCTCTGTTGAG
SB8_F	TTCCACAGACTGATCCAAGGCCACCATGGTGAGCAAGGGCGAG
SB8_R	GTCATTTCGAACCCCAGAGTCCCGCTTACTTGTACAGCTCGTCCA
SB9_F	AACAGAGATTTCAACGAATGCCACCATGGCCATCATCAAGGAG
SB9_R	CCTCGACGTCACCGCATGTTAGCAGACTTCCTCTGCCCTCAGATCCCTTGTACAGCTCGT
SB7_F	TATCTTTACATGTAGCTTGTGCAT
SB7_R	ATTCGTTGAAATCTCTGTTGAG
SB10_F	GGTGGCCTTGGATCAGTCTG
SB10_R	GCGGGACTCTGGGGTTCGAAAT
SB11_F	AGCGAGTCAGTGAGCGAGGAAGCGGAAGATAGATCATGGAGATAATTAAAATGATAACCA
SB11_R	AGCTCCTCGCCCTTGCTCACCATGGTGGCGGTTTCGGACCGAGATCC
SB12_F	CGCGTGATGAACTTCGAG
SB12_R	TTCAGCCTCTGCTTGATCTC
SB13_F	TGGAAAATGTCCGATTCTACG
SB13_R	GCCCATCATTGAACTGTGC

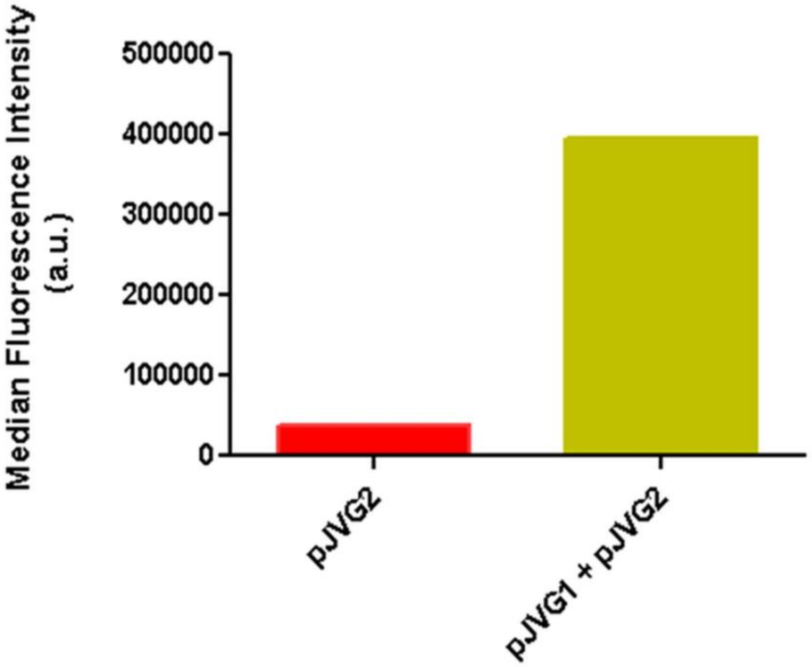

Supplementary Figure S1:

Histogram plot showing the median fluorescence intensity of the mCherry signal in samples as shown in Figure 4E.
